# Delayed diagnosis of extrapulmonary tuberculosis in a 32-year-old man with knee pain

**DOI:** 10.1259/bjrcr.20150258

**Published:** 2016-02-28

**Authors:** Francesco Agnello, Massimo Galia, Giuliana Guadagnino, Viola Ricceri, Federico Midiri, Claudia Colomba

**Affiliations:** ^1^ Section of Radiological Sciences, DIBIMED, University of Palermo, Palermo, Italy; ^2^ Section of Infectious Diseases, University of Palermo, Palermo, Italy; ^3^ School of Medicine, University of Palermo, Palermo, Italy

## Abstract

A 32-year-old Bangladeshi male was admitted at our emergency department for trauma of the left knee. The radiographs showed absence of fracture, and presence of an indeterminate oval lucency in the proximal tibia. Further examinations were suggested, but the patient refused. 6 months later, the patient re-presented at our emergency department. A CT scan showed progression of musculoskeletal involvement and spread to the liver. This case underlines the importance of considering tuberculosis in the differential diagnosis of indeterminate bone lesions in immigrant patients.

## Summary

A 32-year-old Bangladeshi male was admitted at our emergency department for trauma of the left knee. The radiographs showed absence of fracture, and presence of an indeterminate oval lucency in the proximal tibia. Further examinations were suggested, but the patient refused. 6 months later, the patient re-presented at our emergency department. A CT scan showed progression of musculoskeletal involvement and spread to the liver. This case underlines the importance of considering tuberculosis in the differential diagnosis of indeterminate bone lesions in immigrant patients.

## Clinical presentation and imaging findings

**Figure 1.**
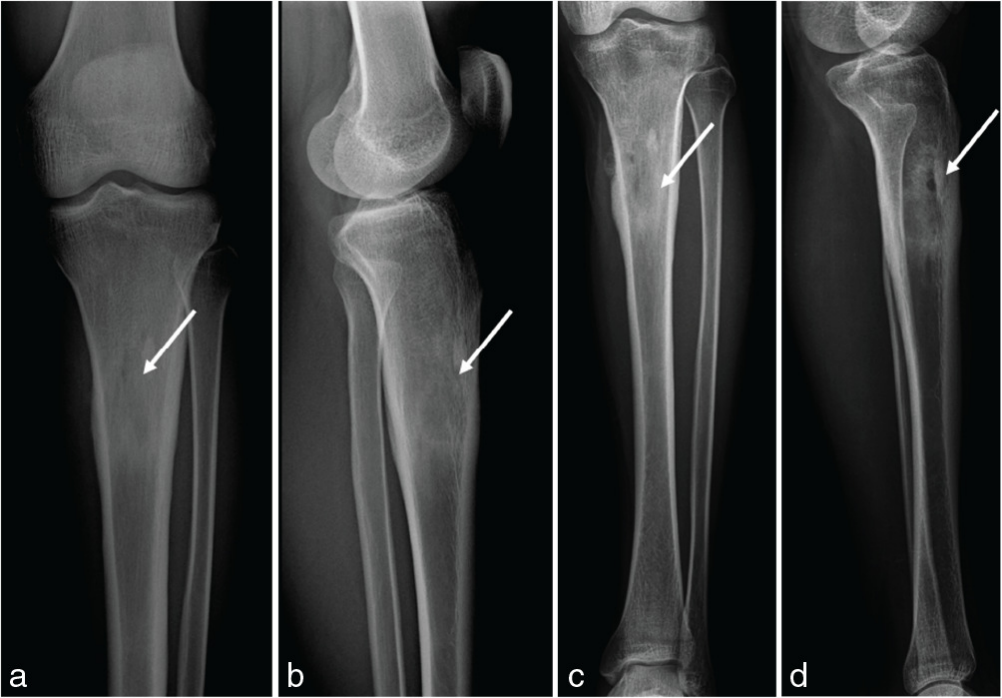
(a, b) Radiographs of the left knee show a well-defined oval lucency in the medulla of the proximal tibial metaphysis (arrows). (c, d) On radiographs of the left tibia obtained 6 months later, the lesion appeared larger, ill-defined and predominantly sclerotic. Also note a small internal lytic area and a smooth periosteal reaction along the medial tibia cortex.

**Figure 2.**
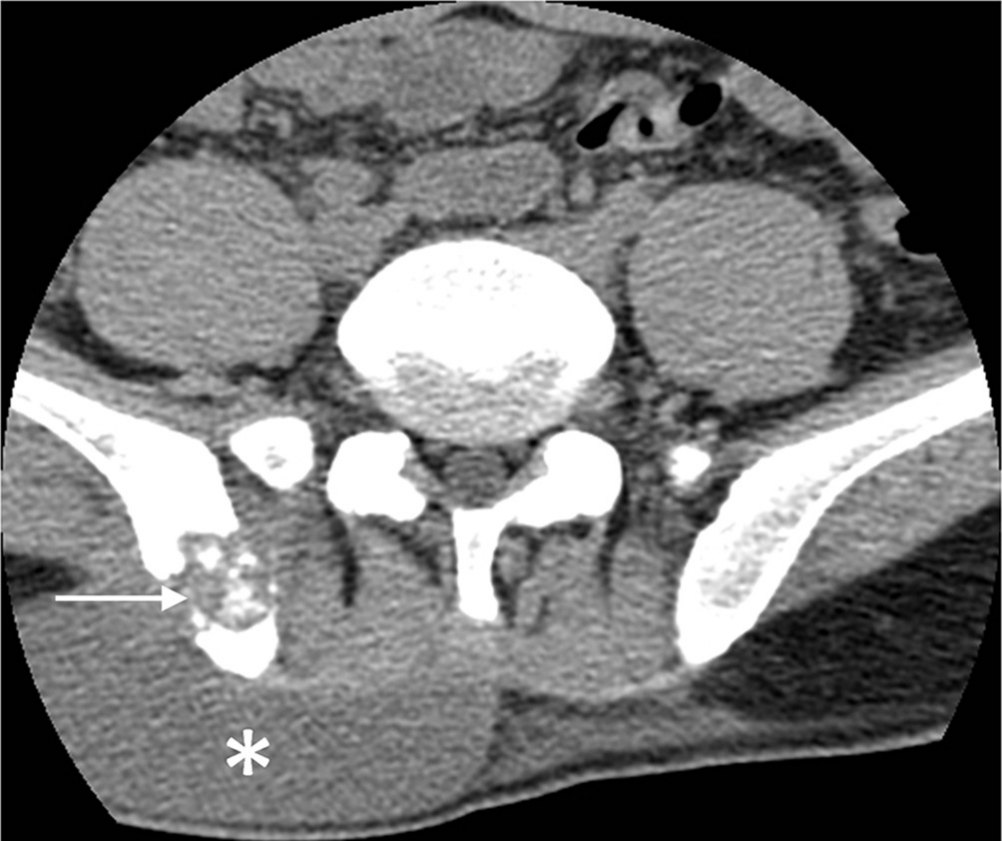
CT scan shows a lytic lesion in the right iliac bone (arrow) in contiguity with a large abscess in the superficial tissue of the posterolateral right abdominal wall (asterisk).

**Figure 3.**
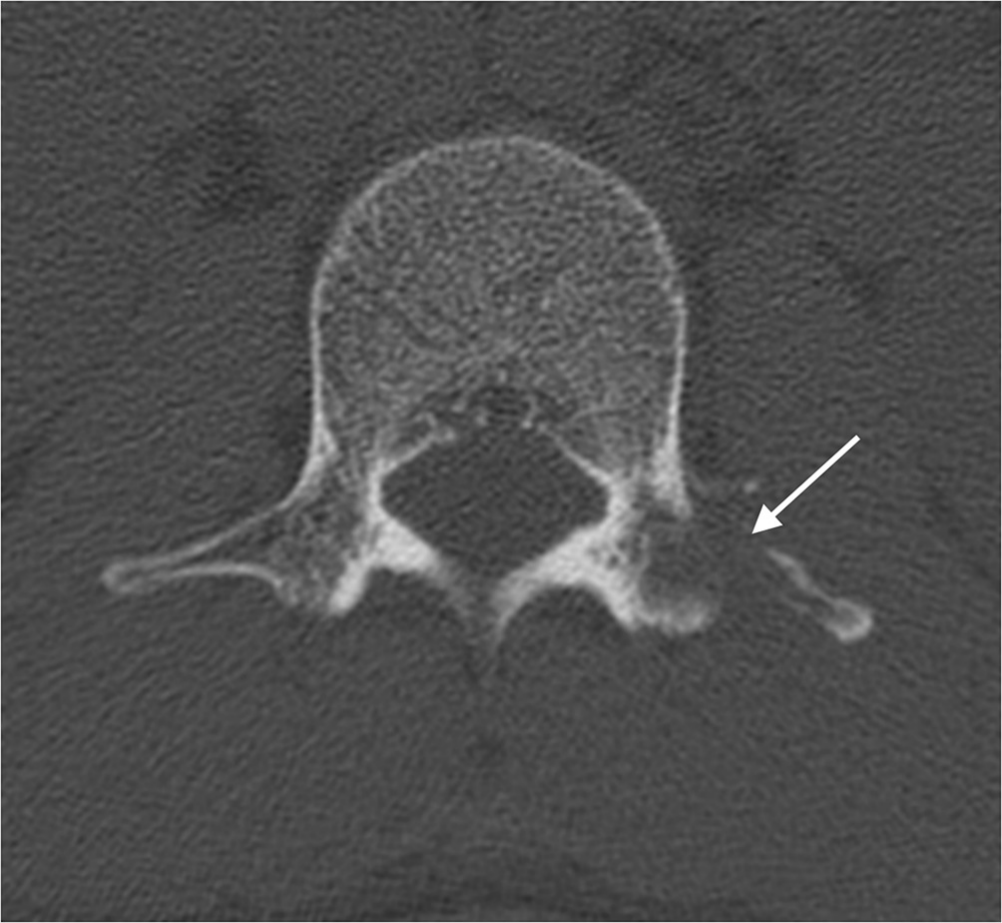
CT scan shows a lytic lesion of the left L1 pedicle (arrow).

A 32-year-old Bangladeshi male was admitted at our emergency department for trauma of the left knee. He had immigrated to Italy from Bangladesh approximately 8 years ago. Physical examination revealed tenderness of the knee but no motion limitation. He had no cough, dyspnoea or fever. The radiographs of the left knee showed a well-defined, oval lucency in the medulla of the proximal tibial metaphysis with surrounding sclerosis (Figure 1a,b). There was no fracture. Further examinations were suggested, but the patient refused. 6 months later, the patient re-presented at our emergency department complaining of intense low back pain, fever of up to 37.5 °C and weight loss over the previous 20 days. Physical examination showed a large right paravertebral mass at the lumbosacral level, hepatomegaly and enlarged cervical and axillary nodes. Neurological symptoms and signs were absent. Laboratory test revealed increased white blood cell count (13,200 mmc^–1^). Test results for human immunodeficiency virus (HIV) were negative. A CT scan of the lumbosacral spine showed a large abscess in the superficial soft tissue of the posterolateral right abdominal wall at the L4–S2 level, in contiguity with a lytic lesion of the right iliac bone (Figure 2). The bone lesion showed cortical destruction and scattered calcifications. A second large, multiloculated abscess extended from the pre-sacral space to the left gluteal muscles and was associated with bone destruction of S5. A lytic lesion with cortical destruction was also noted in the left L1 pedicle (Figure 3). The chest radiograph was within normal limits. MRI of the lumbosacral spine was not performed. On further questioning, the patient revealed a history of mediastinal tuberculosis 2 years earlier. Thus, on the basis of radiological findings and patient history, a diagnosis of extrapulmonary tuberculosis was suspected. The QuantiFERON-TB Gold test supported our hypothesis and antituberculosis therapy was started.

**Figure 4.**
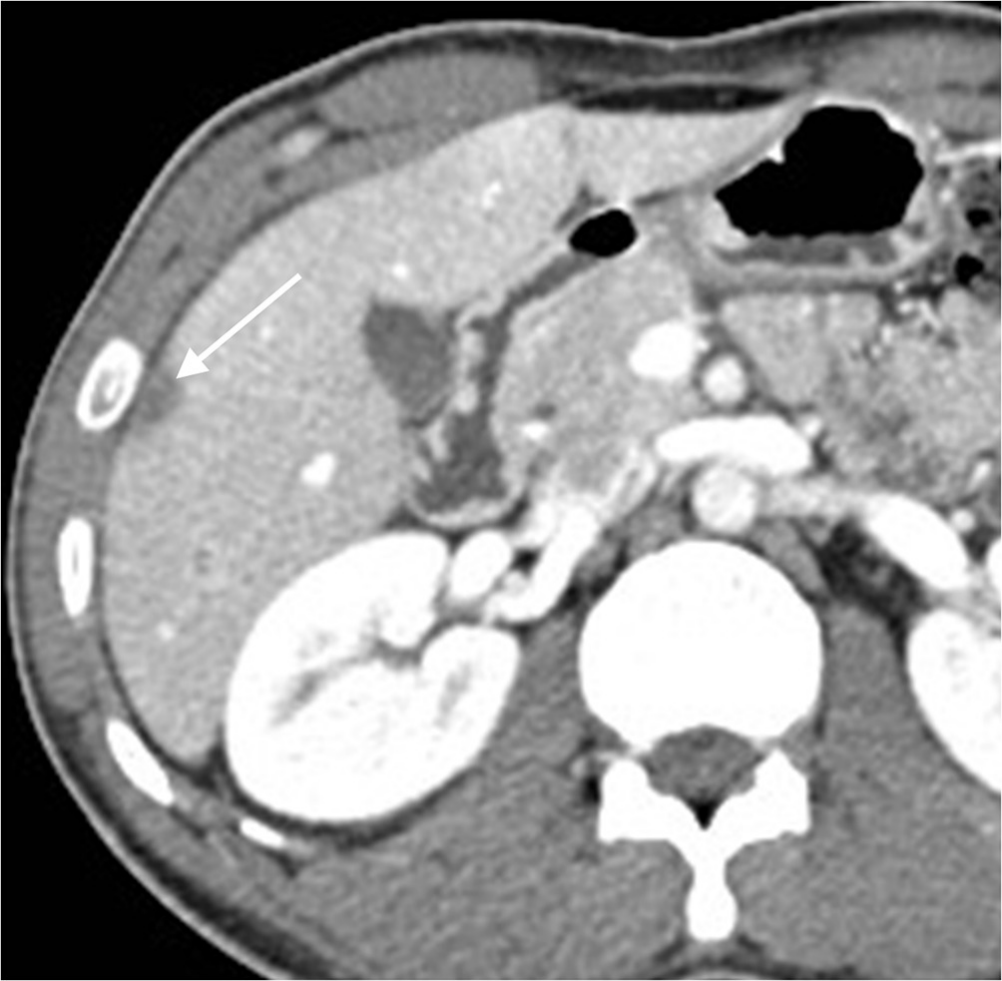
Contrast-enhanced CT scan shows a hypoattenuating, non-enhancing nodule in the liver, compatible with macronodular hepatic tuberculosis (arrow).

Sputum and urine cultures were negative. Subsequently, a CT scan of the chest, abdomen and pelvis with intravenous contrast was planned to search for other tuberculosis locations. It showed multiple, hypoattenuating, 5–15 mm in diameter, non-enhancing nodules in the liver compatible with macronodular hepatic tuberculosis (Figure 4). In spite of antituberculosis therapy, the abscesses appeared larger. There was no abdominal lymphadenopathy or other abdominal abnormalities. The CT scan of the chest did not show any significant findings. On radiographs of the left tibia, the previously noted bone lesion became larger, ill-defined and predominantly sclerotic, with a small internal lytic area and a smooth periosteal reaction along the medial tibial cortex (Figure 1c,d).

## Treatment

The patient was treated with four-drug antituberculosis therapy in accordance with World Health Organization guidelines. The therapy consisted of a daily regimen of 600 mg of rifampin, 300 mg of isoniazid, 1500 mg of pyrazinamide and 1500 mg of ethambutol.^1^ The therapy was effective and the general clinical conditions showed a significant and progressive improvement. The two abscesses, however, were unresponsive to medical treatment. Thus, an open drainage procedure was planned and 900 cc of caseating granulomatous material mixed with blood was drained. Subsequent cultures and polymerase chain reaction were positive for *Mycobacterium tuberculosis.* On day 26, the patient was discharged. The four-drug antituberculosis therapy was continued for the next 12 months.

## Outcome

The patient was followed for 36 months without evidence of recurrence. A follow-up CT scan obtained 18 months after the start of therapy showed disappearance of all the lesions.

## Discussion

The majority of cases of tuberculosis reported in Western countries occur in immigrants from endemic areas. Predisposing factors include poverty, overcrowding, malnutrition, alcoholism, drug abuse, diabetes mellitus, immunosuppressive treatment and HIV infection.^2^ The symptoms are sometimes non-specific, and disease progression can be slow. In addition, more frequent disorders such as metastatic diseases and pyogenic infections can display similar imaging findings.^3^ Because imaging plays a crucial role in identifying both the pulmonary and extrapulmonary tuberculosis, radiologists need to be familiar with tuberculosis morphologic and clinical manifestations.

Herein, we report a case of extrapulmonary tuberculosis with musculoskeletal and hepatic involvement. The initial radiography of the left knee showed a lucency in the proximal tibia. The presence of well-defined margins and marginal sclerosis suggested a benign process. Multiple potential causes could be considered for this lesion, ranging from infectious processes to benign tumours. A diagnosis of tuberculosis, however, was not suspected by the radiologist and the emergency physician, and the patient was discharged without antituberculosis therapy. As the disease progressed, the patient re-presented at our emergency department with more extensive musculoskeletal involvement and spread to the liver. Notably, the tibial lesion in this patient was initially considered an incidental finding.

Musculoskeletal involvement is seen in 1–3% of patients with tuberculosis.^3^ Active thoracic tuberculosis is absent in more than 50% of patients.^3^ The spine (especially the lower thoracic and upper lumbar levels) constitutes the most common site.^3^ Typically, the disease starts in the anterior part of the vertebral body and spreads into adjacent intervertebral disks (resulting in the involvement of multiple vertebral bodies) and paravertebral muscles. A prompt diagnosis is crucial to prevent potential complications such as compression fracture, gibbus and extension into the spinal canal with permanent neurological deficits. In our case, however, the disease involved the sacrum and left L1 pedicle, and was confined to a single vertebral body. These findings are frequently observed in non-white patients and reflect differences in tuberculosis appearance among white and non-white patients.^4^ Moreover, owing to its posterior location and absence of disk involvement, vertebral body involvement may resemble bone metastases.^5^ In our case, however, the presence of soft-tissue abscesses strongly suggested an infectious process. The abscesses are a consequence of bone disease extension into the contiguous soft tissues. Less common locations of musculoskeletal tuberculosis include the femur and the tibia. Tuberculous osteomyelitis is usually associated with tuberculous arthritis. An isolated tuberculous osteomyelitis, as observed in our case, is less common.^5^

Tuberculous hepatic involvement occurs owing to hematogenous dissemination. Two types have been described: the micronodular (miliary) hepatic and the macronodular (tuberculoma) hepatic tuberculosis.^3^ The former is characterized by multiple tiny (0.5–2 mm) nodules scattered into the liver.^3^ The latter is less common and is characterized by multiple 1–3 cm hepatic nodules.^3^ Differential diagnosis of hepatic tuberculosis includes metastatic disease, fungal infection and lymphoma.

In conclusion, this case underlines the importance of considering tuberculosis in the differential diagnosis of indeterminate bone lesions in immigrant patients. Radiologists need to be familiar with tuberculosis epidemiology and imaging appearance. An accurate diagnosis of tuberculosis requires an interdisciplinary approach, which correlates clinical, laboratory and radiological findings.

## Learning points

1. Tuberculosis should be considered in the differential diagnosis of indeterminate bone lesions in immigrant patients.
2. Tuberculosis appearance can be different among white and non-white patients.

## Consent

We confirm that the case report had been sufficiently anonymized to protect patient identity and informed consent could not be obtained despite exhaustive efforts.
